# Supplementary material for: Hepatitis B Co-Infection Has Limited Impact on Liver Stiffness Regression in Chronic Hepatitis C Patients Treated with Direct-Acting Antivirals
Source: Viruses. 2022 Apr 10;14(4):786. doi: 10.3390/v14040786 (PMC9024676; doi:10.3390/v14040786)
Supplement: Supplementary file 1 [file viruses-14-00786-s001.zip › viruses-1649390-supplementary.pdf]

viruses

Supplementary Material

Table S1. Pretherapy demographic comparison between patients with and without HBV coinfection.

Variables	All (N = 906)	Mono-HCV or B+C coinfection		P value
		Mono-C (N = 854)	B+C (N = 52) *	
Age (years)	63.2 ± (11.73)	63.28 ± (11.73)	61.87 ± (11.74)	0.317
Male, n (%)	360 (39.735%)	332 (38.876%)	28 (53.846%)	0.032
Genotype, n (%)				
Non-1	328 (36.203%)	307 (35.948%)	21 (40.385%)	0.518
1	578 (63.797%)	547 (64.052%)	31 (59.615%)	
HCV RNA (Log_{10}IU/ml)	6.192 (1.98~10.63)	6.188 (1.98~10.63)	6.219 (3.7~7.04)	0.273
≥ 6	535 (59.181%)	505 (59.203%)	30 (58.824%)	0.957
< 6	369 (40.819%)	348 (40.797%)	21 (41.176%)	
BMI (kg/m^2)	24.44(15.56~44.82)	24.44 (15.56~44.82)	24.39 (16.8~32.89)	0.479
HbA1c	5.7 (4.3~11.9)	5.7 (4.3~11.9)	5.75 (4.9~11.3)	0.621
≥ 6.5	168 (19.178%)	157 (18.961%)	11 (22.917%)	0.499
< 6.6	708 (80.822%)	671 (81.039%)	37 (77.083%)	
HOMA index	2.11 (0.2~84.13)	2.12 (0.2~84.13)	1.97 (0.38~58.29)	0.386
≥ 2.5	245 (37.462%)	234 (37.987%)	11 (28.947%)	0.264
< 2.5	409 (62.538%)	382 (62.013%)	27 (71.053%)	
Cholesterol (mg/dL)	170.0 (81.0~283.0)	170.0 (81.0~283.0)	176.0 (97.0~230.0)	0.324
≥ 200	134 (17.867%)	127 (17.862%)	7 (17.949%)	0.989
< 200	616 (82.133%)	584 (82.138%)	32 (82.051%)	
Triglyceride (mg/dL)	91.0 (29.0~442.0)	91.0 (29.0~442.0)	91.0 (39.0~299.0)	0.864
≥ 100	297 (39.812%)	282 (39.887%)	15 (38.462%)	0.86
< 100	449 (60.188%)	425 (60.113%)	24 (61.538%)	
LDL (mg/dL)	99.0 (31.0~400.0)	99.0 (31.0~400.0)	109.0 (36.0~152.0)	0.315
≥ 130	123 (16.757%)	116 (16.643%)	7 (18.919%)	0.718
< 130	611 (83.243%)	581 (83.357%)	30 (81.081%)	
ALT (U/L)	56.5 (5.0~895.0)	57.0 (5.0~895.0)	56.0 (12.0~324.0)	0.514
< 1xULN, n (%)	262 (28.918%)	248 (29.04%)	14 (26.923%)	0.946
1-5x	574 (63.355%)	540 (63.232%)	34 (65.385%)	
≥ 5x	70 (7.726%)	66 (7.728%)	4 (7.692%)	
T-bil. (mg/dL)	0.7 (0.2~6.0)	0.7 (0.2~6.0)	0.8 (0.2~1.7)	0.201
Albumin (g/dL)	4.27 (2.7~5.21)	4.26 (2.7~5.21)	4.325 (2.88~4.8)	0.202
INR	1.1 (0.9~3.0)	1.1 (0.9~3.0)	1.1 (0.9~1.4)	0.128
AFP (ng/mL)	4.2 (1.0~2137.3)	4.3 (1.0~2137.3)	3.45 (2.0~205.0)	0.127
Platelet (10^3/uL)	165.0 (20~404)	164.0 (20~404)	179.5 (42~338)	0.697
FIB-4 score	2.74 (0.29~38.29)	2.765 (0.29~38.29)	2.37 (0.41~16.38)	0.393
FIB4 < 1.45	151 (16.667%)	140 (16.393%)	11 (21.154%)	0.667
1.45 ≤ FIB4 < 3.25	374 (41.28%)	354 (41.452%)	20 (38.462%)	
3.25 ≤ FIB4	381 (42.053%)	360 (42.155%)	21 (40.385%)	
LSM	10.1 (2.8~75.0)	10.2 (2.8~75.0)	8.15 (3.6~48.0)	0.091
LSM < 7.1	296 (32.671%)	273 (31.967%)	23 (44.231%)	0.217
7.1 ≤ LSM < 9.5	137 (15.121%)	128 (14.988%)	9 (17.308%)	
9.5 ≤ LSM < 12.5	173 (19.095%)	166 (19.438%)	7 (13.462%)	
12.5 ≤ LSM	300 (33.113%)	287 (33.607%)	13 (25.0%)	
End of treatment ALT	19.0 (5.0~635.0)	18.0 (5.0~248.0)	22.0 (6.0~635.0)	0.031

AFP, alpha-fetoprotein; ALT, alanine aminotransferase; AST, aspartate aminotransferase; BMI, body mass index; DAA, direct antiviral agents; FIB4, The Fibrosis-4 Index; HbA1c, glycohemoglobin; HBV, hepatitis B virus; HCV, hepatitis C virus; HOMA, Homeostatic Model Assessment for Insulin Resistance; INR, international normalized ratio; LDL: low-density lipoprotein; LSM: liver stiffness measurement; T-bil.: total bilirubin; ULN, upper limit of normal; x, times. *HBeAg status is available in 37/52 patients in coinfection group; all 37 patients were HBeAg negative.

Table S2. Liver stiffness measurements change in Mono HCV and HBV/HCV-co-infection.

Subgroups	Liver stiffness measurements (Kpa)		P value
	Pre-treatment	Post-treatment	
All patients (N = 906)	10.15 (2.8–75.0)	6.9 (2.6-75.0)	<0.001
HCV mono-infection (N = 854)	10.2 (2.8–75.0)	6.9 (2.6–75.0)	<0.001
LSM < 7.1(N = 273)	5.3 (2.8–7.0)	4.6 (2.6–11.7)	<0.001
7.1 ≤ LSM < 9.5 (N = 128)	8.0 (7.1–9.4)	6.1 (3.8–36.3)	<0.001
9.5 ≤ LSM < 12.5 (N = 166)	10.8 (9.5–12.4)	7.6 (3.5–21.1)	<0.001
12.5≤ LSM (N = 287)	22.1 (12.5–75.0)	16.0 (4.9–75.0)	<0.001
HBV co-infection (N = 52)	8.3 (3.6–48.0)	6.3 (3.3–46.4)	<0.001
LSM < 7.1(N = 23)	5.4 (3.6–7.0)	4.8 (3.3–7.9)	0.211
7.1 ≤ LSM < 9.5 (N = 9)	8.7 (7.7–9.1)	6.9 (3.3–11.6)	0.164
9.5 ≤ LSM < 12.5 (N = 7)	11.1 (10.1–12.0)	9.1 (6.5–14.3)	0.219
12.5 ≤ LSM (N = 13)	17.75 (13.3–48.0)	13.05 (4.9–46.4)	0.002

HBV, hepatitis B virus; HCV, hepatitis C virus; LSM, liver stiffness measurement.

Table S3. Demographic features before start of DAA therapy between HBV/HCV coinfected patients with pre-DAA viremia ≥ 2000 vs. < 2000IU/mL.

Variables	HBV infection status			P value
	Mono-C (N = 156)	DNA < 2000 IU/mL (N = 42)	DNA ≥ 2000IU/mL (N = 7)	
HBV DNA(IU/ml)		27.0 (0.0~1160.0)	11492.0 (2525.0~61551.0)	
Age (years)	62.85 (31.6~91.9)	62.35 (26.4~84.4)	61.8 (48.2~80.0)	0.716
Male, n (%)	84 (53.846%)	21 (50.0%)	5 (71.429%)	0.572
Cirrhosis by echo	40 (25.641%)	12 (28.571%)	1 (14.286%)	0.721
Genotype, n (%)				0.220
Non-1	46 (29.487%)	18 (42.857%)	3 (42.857%)	
1	110 (70.513%)	24 (57.143%)	4 (57.143%)	
HCV RNA (Log_{10}IU/ml)	6.21 (3.23~7.29)	6.22 (3.70~7.04)	6.25 (5.11~6.78)	0.713
BMI (kg/m2)	24.47 (17.07~37.50)	24.39 (16.80~31.11)	22.78 (18.83~32.89)	0.665
HbA1c	5.7 (4.6~11.9)	5.7 (4.9~11.3)	5.8 (5.2~9.9)	0.933
HOMA index	2.16 (0.48~19.71)	1.925 (0.38~58.29)	1.98 (0.88~7.34)	0.44
Cholesterol (mg/dL)	166.0 (115.0~283.0)	170.0 (97.0~230.0)	198.0 (173.0~215.0)	0.092
Triglyceride (mg/dL)	94.0 (34.0~275.0)	92.0 (47.0~299.0)	89.0 (39.0~206.0)	0.496
LDL (mg/dL)	102.0 (47.0~191.0)	102.0 (36.0~152.0)	120.0 (100.0~143.0)	0.159
ALT (U/L)	47.0 (8.0~273.0)	56.0 (15.0~324.0)	42.0 (14.0~263.0)	0.722
Post Treatment ALT (U/L)	18.0 (6.0~173.0)	21.5 (6.0~635.0)	29.0 (11.0~79.0)	0.081
T-bil. (mg/dL)	0.7 (0.2~3.4)	0.7 (0.2~1.7)	0.9 (0.8~1.4)	0.082
Albumin (g/dL)	4.28 (3.39~5.15)	4.335 (3.54~4.8)	4.34 (4.06~4.7)	0.525
INR	1.1 (0.9~1.6)	1.1 (0.9~1.4)	1.1 (1.0~1.3)	0.158
AFP (ng/mL)	3.65 (1.0~248.1)	3.45 (2.0~205.0)	2.6 (2.2~5.6)	0.369
Platelet (103/uL)	178.0 (29.0~384.0)	165.5 (42.0~338.0)	199.0 (189.0~244.0)	0.035
FIB-4 score	2.34 (0.29~20.18)	2.445 (0.41~16.38)	2.14 (1.07~3.61)	0.391
LSM	7.7 (3.4~52.3)	8.15 (3.6~48.0)	5.8 (3.7~17.3)	0.726
LSM change	−1.65 (−26.4~11.6)	−0.8 (−30.9~2.5)	−0.6 (−3.6~3.8)	0.234
Post treatment LSM	5.9 (2.8~63.9)	6.2 (3.3~46.4)	5.8 (3.5~14.3)	0.963

AFP, alpha-fetoprotein; ALT, alanine aminotransferase; AST, aspartate aminotransferase; BMI, body mass index; DAA, direct antiviral agents; FIB4: The Fibrosis-4 Index; HbA1c, glycohemoglobin; HBV, hepatitis B virus; HCV, hepatitis C virus; HOMA, Homeostatic Model Assessment for Insulin Resistance; INR, international normalized ratio; LDL: low-density lipoprotein; LSM: liver stiffness measurement; T-bil.: total bilirubin; ULN, upper limit of normal; x, times.

Table S4. Post treatment demographic comparison after PSM (1:3 match, n = 208).

Variables	All (N = 208)	Mono HCV or B+C coinfection		P value
		Mono-C (N = 156, 75%)	B+C (N = 52, 25%)	
Post treat FIB-4 score	2.185 (0.41~15.57)	2.235 (0.49~15.57)	2.1 (0.41~6.28)	0.712
FIB4 < 1.45	2.19 (0.41~15.6)	2.21 (0.49~15.6)	2.11 (0.41~6.28)	0.622
1.45 ≤ FIB4 < 3.25	42 (21.212%)	32 (21.477%)	10 (20.408%)	0.226
3.25 < FIB4	109 (55.051%)	86 (57.718%)	23 (46.939%)	
FIB4 change	−0.13 (−9.74~3.25)	−0.07 (−6.22~3.25)	−0.28 (−9.74~1.39)	0.109

Post treat LSM	6.0 (2.8~63.9)	5.9 (2.8~63.9)	6.2 (3.3~46.4)	0.763
LSM < 7.1	127 (61.058%)	98 (62.821%)	29 (55.769%)	0.520
7.1 ≤ LSM < 9.5	25 (12.019%)	19 (12.179%)	6 (11.538%)	
9.5 ≤ LSM < 12.5	21 (10.096%)	13 (8.333%)	8 (15.385%)	
12.5 ≤ LSM	35 (16.827%)	26 (16.667%)	9 (17.308%)	

ALT, alanine aminotransferase; HBV, hepatitis B virus; HCV, hepatitis C virus; LSM, liver stiffness measurement;.

Table S5. Patients received NUC during DAAs.

			**Pre-DAA profile**							**Hepatitis flare event**				**Post DAA**		
**Case**	**Age**	**Gender**	**HCV GT**	**HCV RNA (Log$_{10}$IU/ml)**	**HBV DNA (IU/ml)**	**LC**	**ALT (U/L)**	**FIB-4**	**LSM (kPa)**	**onset#**	**NUCs**	**HBV DNA (IU/ml)**	**ALT* (U/L)**	**LSM (kPa)**	**ALT (U/L)**	**Outcome**
#1	52	M	1a	6.5	894	No	46	0.96	7	Wk 8	ETV	$2.88*10^{9}$	1584	7.9	635	Recovery in 3mos
#2	48	M	1b	6.36	58406	No	14	1.07	5.8	Wk 6	ETV	$2.61*10^{6}$	945	5.8	36	Recovery in 1mos

ETV: entecavir; GT: genotype; HBV, hepatitis B virus; HCC, hepatic cellular carcinoma HCV, hepatitis C virus; LC: cirrhosisLSM, liver stiffness measurement; M: Male; mos: months; Nuc: nucleos(t)ide analogue; Wk: week; *: peak level; #: counted from start of DAA treatment.

Table S6. Patients develop hepatic decompensation after SVR.

			**Pre-DAA profile**							Post DAA	Decompensation event		
**Case**	**Age**	**Gender**	**HCV GT**	**HCV RNA (Log$_{10}$IU/ml)**	**HBV DNA (IU/ml)**	**LC**	ALT (U/L)	FIB-4	LSM (kPa)	LSM (kPa)	onset	events	Outcome
#1	60	F	1b	6.36	Undetectable	Yes	102	8.02	48	36.3	3 years after EOT	New esophageal varices	Stable after endoscopic treatment
#2	73	M	1b	4.62	Undetectable	Yes	80	16.38	17.6	11.8	6 months after EOT	New esophageal varices	Stable after endoscopic treatment
#3	47	F	1b	5.47	160	Yes	38	4.33	28.4	10.8	3.5 years after EOT	Liver failure	Expired

EOT: end of treat; ETV: entecavir; GT: genotype; HBV, hepatitis B virus; HCC, hepatic cellular carcinoma HCV, hepatitis C virus; LC: cirrhosisLSM, liver stiffness measurement; M: Male; mos: months; Nuc: nucleos(t)ide analogue; Wk: week; *: peak level; #: counted from start of DAA treatment.

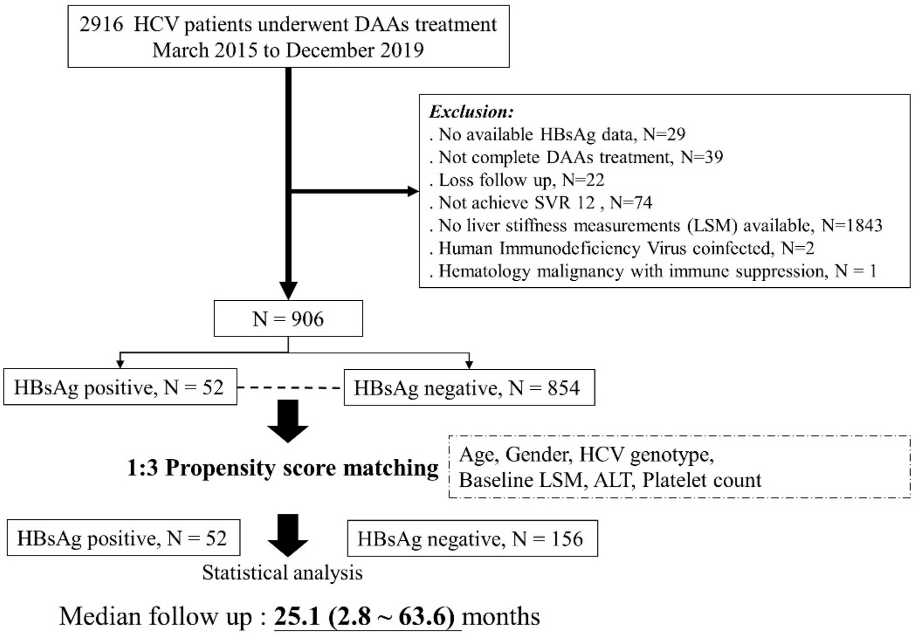

Figure S1. Flowchart of patient enrolment.
